# Microleakage Evaluation of Adhesive Systems Following Pulp Chamber Irrigation with Sodium Hypochlorite

**DOI:** 10.5681/joddd.2014.004

**Published:** 2014-03-05

**Authors:** Mohammad Javad Moghaddas, Horieh Moosavi, Marjaneh Ghavamnasiri

**Affiliations:** ^1^Associate Professor, Dental Materials Research Center, Department of Operative Dentistry, Mashhad Dental School, Mashhad University of Medical Sciences, Mashhad, Iran; ^2^Associate Professor, Dental Materials Research Center, Department of Operative Dentistry, Mashhad Dental School, Mashhad University of Medical Sciences, Mashhad, Iran; ^3^Professor, Dental Research Center, Department of Operative Dentistry, Mashhad Dental School, Mashhad University of Medical Sciences, Mashhad, Iran

**Keywords:** Adhesive system, sodium hypochlorite, microleakage

## Abstract

***Background and aims.*** This in vitro study evaluated the effect of delaying composite resin restorative procedures bonded with total-etch and self-etch adhesive systems on microleakage following root canal irrigation with sodium hy-pochlorite (NaOCl) solution.

***Materials and methods.*** The roofs of pulp chambers and roots (1&ndash;2 mm below furcation) of 40 human first molar teeth were cut and pulp tissues completely removed. The teeth were randomly divided into two main groups (n = 20). Group E (experimental) was irrigated with 5% NaOCl and group C (control) was left untreated. For the experimental group, after obturation of root canals with gutta-percha and sealing the cavity with Cavit, the specimens were stored in artificialsaliva for two weeks. Then each group was divided into two subgroups according to the total-etch or self-etch adhesive application protocol: Scotchbond Multi-Purpose and Clearfil SE Bond. The specimens were restored with composite resin using each bonding agent: Z250 and Clearfil Photo Core, respectively. Fluid filtration method was used for evaluation of microleakage. Data was analyzed using two-way ANOVA ( α= 0.05).

***Results.*** Two types of dentin adhesive systems showed no statistically significant differences in microleakage (P = 0.77). NaOCl-treated groups demonstrated significantly higher microleakage values compared to the non-NaOCl-treated groups (P= 0.001). The interaction between the two factors was not significant (P = 0.78).

***Conclusion.*** Differences in inlay temperature had no effect on microleakage. CAD/CAM inlays had lower cement thick-ness than laboratory-made inlays, but this was not related to their microleakage.

## Introduction

Use of adhesive systems offers many advantages over the use of nonadhesive materials. Lack of sealing and proper adhesion between the final restoration and tooth structure may adversely affect the results of root canal treatment. Therefore, the coronal seal is likely to be a factor in the long-term success of root canal treatment.^1^ None of the current dental materials provides a hermetic and leak-proof seal.^2,3^ There are numerous commercial bonding agent systems with different strategies, which are categorized by "generations" with total-etch being described as the fourth and fifth generation and self-etch being described as the sixth and seventh generation bonding systems. These bonding strategies might behave differently on dentin surfaces within pulp chambers altered by irrigants and during root canal treatment.^4^ On the other hand, pulp chamber walls present complexity because they consist of predentin, secondary and tertiary dentin, and a large number of dentinal tubules.^5^ Investigation of adhesion of these materials to pulp chamber dentinal walls has not been reported extensively. Root canal irrigation has been considered the best way for eliminating the remaining soft tissues or dentinal debris after instrumentation. To this end, sodium hypochlorite (NaOCl) solution remains the most widely recommended irrigant in endodontics. NaOCl is a nonspecific proteolytic agent that dissolves necrotic tissue remnants and organic compounds with excellent antimicrobial potency.^6^ NaOCl has also been frequently applied for collagen removal with controversial results.^7^ Some previous studies have shown that dentin exposed to NaOCl exhibits lower resin bond strength and higher microleakage compared to untreated dentin. This interference is mainly because of the effect of the residual NaOCl on resin polymerization and the interaction of different adhesive systems to the oxidizing effect of sodium hypochlorite.^8,9^ The residual NaOCl could be neutralized by the application of sodium ascorbate to the oxidized dentin, which acts as a reducing agent, restoring the redox potential of dentin and converting the microenvironment of the dentin from an oxidized substrate to a reduced substrate, thus facilitating complete polymerization.^8-10^ Lai et al^8^ demonstrated that a reduction in bond strength caused by NaOCl could be reversed by a one-minute application of a neutral biocompatible antioxidant: 10% sodium ascorbate. End products formed by the reaction between sodium ascorbate and sodium hypochlorite are oxalic acid and L-threonic acid, both of which are water soluble. Although in vitro studies have shown the negative effects of root canal irrigation with NaOCl on microleakage of composite resin, the effect of delaying the restoration of pulp chamber with composite resin following root canal irrigation with NaOCl on microleakage has not been established. Therefore, the aim of this in vitro study was to evaluate the effect of delaying restorative procedures to bond composite resin to pulp chamber dentin for two weeks on microleakage using two adhesive systems following root canal irrigation with 5% NaOCl. The null hypotheses of this study regarding microleakage were: There is no significant difference between groups treated and untreated with 5% NaOCl and no significant difference between the two adhe-sive systems.

## Materials and Methods

Forty sound molar teeth with fully developed apices were used within three months following extraction. After disinfection of the teeth with 0.2% Thymol solution for 24 hours,^11^ they were also thoroughly scaled using curettes to remove calculus and remaining tissue tags. The roof of the pulp chamber was removed and the roots were cut 1–2 mm below the furcation using a diamond disk (Tiz Kavan Co, Tehran, Iran) mounted on a low-speed handpiece. The pulp tissue was removed carefully with an excavator and endodontic instruments. Canal orifices were widened with #2 and #3 Gates-Glidden drills (L.D. Caulk Division, Dentsply International, Inc, Milifored, Del.). The specimens were divided into two groups (n = 20). Group E (experimental) was irrigated with 5% sodium hypocholorite (HYPOSET, USP Hygienes, India) for five minutes and then rinsed with water, and group C (control) was left untreated. NaOCl was used for 5 minutes based on previous research and estimation of the approximate time for the treatment of a molar tooth during canal preparation with NaOCl.^12^ The canal orifices were obturated with a gutta-percha master cone #35–40 (DiaDent, Choongchong Buk DO, Korea) without a root canal sealer. The access cavities were sealed with Cavit (3M/ESPE, Seefeld, Germany). The teeth were stored in artificial salivawith a chemical composition of KCl (0.4 g/L), NaCl (0.4 g/L), CaCl_2_.2H_2_O (0.906 g/L), NaH_2_PO_4_.2H_2_O (0.690 g/L), Na_2_S.9H_2_O (0.005 g/L), and urea (1 g/L) with pH adjusted to 7.03, for two weeks.^13^ Then each group was randomly divided into two subgroups (n = 10), according to the application of a total-etch adhesive: Scotchbond Multi-Purpose (3M ESPE Dental Products, St Paul, Minn.) groups ES and CS; and a self-etch adhesive: Clearfil SE Bond (Kuraray Medical Inc., Japan) groups EC and CC. Pulp chambers of all the groups were restored with composite resin using each bonding agent: Z250 (3M ESPE), and Clearfil Photo Core (Kuraray Medical Inc.) according to manufacturers’ directions, and light-cured at 500 mW/cm^2^ (Optilux 500, Demetron-Kerr, Orange, Calif.). All the specimens had approximately a 2-mm-thick layer of restorative material placed into the pulp chamber floor with the incremental technique (Table 1).

**Table 1:** Material names, compositions and restorative procedures used in this study

Restorative Procedure	Composition	Product name
Dentin bonding system		
Etch for 15 s, rinse for15 s, gently dry for 5 s	37% phosphoric acid , water	
Apply one coat of primer		
10 second- air dry 5 s		
Apply one coat of adhesive, light cure 10 s with 500 mW/cm^2^	HEMA, polyalkenoic copolymer, water
20 s apply, gently dry	Bis-GMA, HEMA, photoinitiator	Scotchbond Multi-Purpose (SBMP) (3M)
Apply, gently dry,	Primer-MDP, HEMA, water, light curing catalyst.	Clearfil SE Bond (CSEB) (Kuraray)
10 s light cured	Adhesive-light curing catalyst	
Resin composite		
Apply in 2 mm thickness, 40 s light-cured	Zirconia/silica particles Bis-GMA, UDMA, Bis-EMA	Z250 (3M)
Apply in 2 mm thickness, 40 s light-cured	Silanated silica, silanated barium glass, bisphenol A diglycidylmethacrylate, CQ	Clearfil Photo Core (Kuraray)
HEMA, 2-Hydroxyethyl methacrylate, ; Bis-GMA, bisphenol A glycidyl methacrylate ; MDP, 10-methacryloyloxydecyl dihydrogen phosphate ; UDMA, Urethane dimethacrylate; CQ, Camphorquinone.		

**Figure 1.**
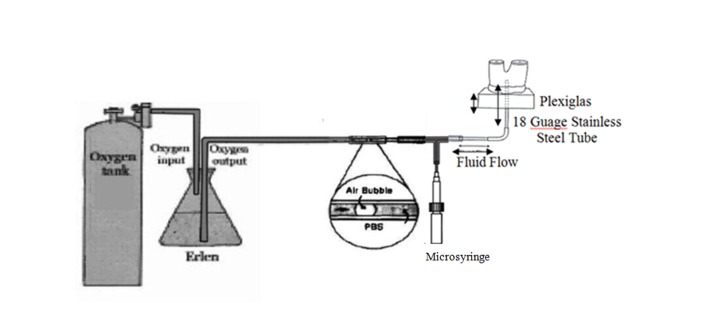
The fluid filtration device.

A probe was used to measure the distance between the floor of the pulp chamber and the occlusal surface; 2 mm was then subtracted from this measurement, and the restorative material was placed into the pulp chamber floor up to that depth. A 2-mm-deep cavity was considered from the coronal end of each pulp chamber segment. This space created a reservoir for the insertion of the protruded metal tubing. The rationale for this statement is that this technique eliminated clogging of the metal tubing and prevented the generation of false negative results.^14^ The cut surfaces of pulp chambers were then cemented on to 2×2×0.7-cm pieces of Plexiglas with a cyanoacrylate adhesive (Zapit, DVA, Corona, Calif.). The pieces of Plexiglas had 18-gauge stainless-steel tubes placed through their centers, ending flush with the upper surfaces. The access opening of the tooth segments were then positioned over the tubes to allow a direct communication between the pulp chamber and the microsyringe system. Each Plexiglas–pulp chamber assembly was attached via polyethylene tubing (Fisher Scientific, Pittsburg, Penn.) to a fluid filtration device. The pulp chambers were filled with water through the 18-gauge needle, taking care to remove all the air bubbles that could be seen through the transparent Plexiglas. Empty root canals beneath the sealing materials were also filled with water to maintain hydration of dentin. The specimens were stored in distilled water at 37°C for 24 hours. The fluid filtration method was used for the quantitative evaluation of leakage. A small 1–2-µL air bubble was introduced to the system with a microsyringe that progressed within a 25-µL micropipette to serve as an indicator of fluid movement. All the tubing pipette and syringe were filled with distilled water under a pressure of 239 cm of H_2_O or 23.4 KPa (Figure 1).

Fluid exited from the pressured reservoir through tubing containing a micropipette connected to the tooth segment. The movement of a tiny air bubble was controlled by the microsyringe, which was proportional to the leakage. Measurements of fluid movement were made at two-minute intervals for eight minutes.^14^ At the end of the leakage method, the results were calculated as a value of liquid pressure (Lp: hydrolytic conductance) and analyzed statistically using two-way ANOVA (α = 0.05).

## Results

The normality of data was confirmed by Kolmogorov-Smirnov test (P > 0.05). Tables Table 2andTable 3 indicate statistical analyses and comparisons (mean ± SD) of adhesive systems in the experimental groups. Two-way ANOVA revealed significant differences between LP (LP = µL/min/cm H_2_O) values of untreated groups and two weeks elapsed after NaOCl-treated groups (P = 0.001) while there were no significant differences between the two adhesive systems regarding LP values (P = 0.77). There was no interaction between the two factors (P = 0.78).

**Table 2:** Means and standard deviations of microleakage in all the experimental groups

Group description	Code	Number	Mean (µL/min/cm H_2_O)	S.D
Without NaOCl+(SBMP)	CS	10	0.000023414	0.00000325
Without NaOCl +(CSEB)	CC	10	0.000022092	0.000013503
With NaOCl+(SBMP)	ES	10	0.00092982	0.00006727
With NaOCl+(CSEB)	EC	10	0.00104781	0.00045533

**Table 3:** Comparison of microleakage in restored specimens with and without NaOCl treatment. (Different letters indicate statistically significant effect.)

Procedure description (Code)	Number	Mean leakage (µL/min/cm H_2_O)	Mean leakage (µL/min/cm H_2_O)	S.D	P
Without 5% NaOCl (CS, CC)	20	0.00022753 ^a^	0.00022753 ^a^	0.00003826	0.001
With 5% NaOCl (ES, EC)	20	0.00093683 ^b^	0.00093683 ^b^	0.00006049	

## Discussion

Many in vitro methods have been used to evaluate the sealing quality of materials in endodontically treated teeth. In this study, fluid filtration technique was used to evaluate the leakage quantitatively. This technique has been suggested to enhance reliability, reproducibility and comparability.^10,15^ In endodontically treated teeth, if adhesive systems are used appropriately, the second line of defense after apical seal against the leakage of bacteria will be created. In addition, the dentin wall of the pulp chamber is not prepared during endodontic procedures and does not have the smear layer; therefore the sealing capacity of adhesive systems depends mostly on the structure of the collagen-rich predentin and the number and permeability of dentinal tubules.^16^ The temporary filling material in this study was Cavit, which was placed with a 4-mm thickness in the pulp chamber. In the present study, final restorative procedures were performed two weeks after root canal therapy. The objectives of treatment were met without significant iatrogenic error, such as perforation. Complex extracoronal restorations may be planned 1–2 weeks after completion of treatment.^17^ There is controversy over delaying the final restoration following root canal therapy. A previous study demonstrated that 50% of 45 root canals were contaminated with *Proteus vulgaris* 42 days after root canal therapy, while another study showed that root canals could be contaminated in less than 30 days after root canal therapy.^18^ Balto et al^19^ suggested that a two-week delay in post space preparation following root canal therapy is needed to allow complete setting of the sealer cement. However, after post space preparation, a permanent restoration should be placed as soon as possible to prevent recontamination of the root canal. The results of the present study indicated that NaOCl-treated groups could increase microleakage values compared to control groups, but there was no significant difference between the two adhesives. Therefore, the first null hypothesis was rejected and the second one was accepted. Existence of microleakage in control groups without surface treatment with both adhesive systems could be attributed to different characteristics of pulp chamber dentin, such as collagen-rich predentin, enlarged tubules, small amounts of intertubular dentin, surface free from the smear layer, and presence of high C-factor. In this study, none of the adhesive systems were capable of completely preventing the penetration of fluid across the bonded interface in the specimens. This is similar to the conclusion of some previous studies and two types of adhesive systems showed similar degree of microleakage.^12,20^ Several studies have also demonstrated comparable microleakage values of total-etch and self-etch systems.^21-23^ The mineralized dentin is porous. It seems that some NaOCl remains within the mineralized subsurface before bonding.^24^ Several previous studies have shown the negative effect of NaOCl on microleakage of composite resin-restored teeth.^10,12,15,19^ Ozturk et al^14^ applied total-etch and self-etch adhesive systems following root canal irrigation with NaOCl and found no significant difference between the two materials at different time intervals.Significantly greater microleakage of resin–dentin interface with both adhesives after NaOCl treatment might be attributed to the oxidizing effects of NaOCl and its reaction byproducts causing inhibition of the interfacial polymerization of adhesives. Some reactive residual free radicals in NaOCl-treated dentin may compete with the propagation of vinyl-free radicals generated during light activation of the adhesive system, resulting in premature chain termination and incomplete polymeraization.^25^ In addition, bonding substrate and the chemical composition of adhesive systems have an important role in microleakage.^26^ NaOCl removes predentin; however, adhesive systems use acidic conditioners that etch the NaOCl-treated surface to expose fresh collagen fibrils for resin-dentin hybridization. It seems that the oxidizing effect of NaOCl could be stable even two weeks after irrigation with NaOCl. Therefore, this study, similar to other studies, recommended applying sodium ascorbate or other antioxidants as a reducing agent or longer delays after NaOCl irrigation and before coronal resin restoration procedure.

## Conclusion

Under the limitations of the present study, both total-etch and self-etch adhesive systems showed similar leakage after NaOCl irrigation. Pulp chamber irrigation with NaOCl resulted in an increase in microleakage values of composite resin restorations, even though two weeks elapsed after irrigation.

## Acknowledgement

Funding for this research was provided by the Research Council of Mashhad University of Medical Sciences, research grant # 85172.
